# Retraction Note: In-hospital cerebrovascular complications following orthotopic liver transplantation: A retrospective study

**DOI:** 10.1186/s12883-021-02175-y

**Published:** 2021-04-10

**Authors:** Li Ling, Xiaoshun He, Jinsheng Zeng, Zhijian Liang

**Affiliations:** 1 Department of Neurology and Stroke Centre, First Affiliated Hospital, Sun Yat-Sen University, No. 58 Zhongshan Road 2, Guangzhou, 510080 PR China; 2 Department of Organ Transplantation, First Affiliated Hospital, Sun Yat-Sen University, No. 58 Zhongshan Road 2, Guangzhou, 510080 PR China; 3 Department of Neurology and Stroke Centre, First Affiliated Hospital, Guangxi Medical University, No. 23, Shuangyong Road, Nanning, 530021 PR China

**Retraction Note: BMC Neurol 8, 52 (2008)**

**https://doi.org/10.1186/1471-2377-8-52**

The authors have retracted this article after concerns were raised with respect to the source of donor organs [1].

This study retrospectively reviewed the data of patients with in-hospital cerebrovascular complications after receiving liver transplants at the Organ Transplantation Center, First Affiliated Hospital of Sun Yat-sen University, from January 1996 to June 2005. The authors have stated that during this period most of the transplanted organs were obtained from executed prisoners; therefore the study reported in this article does not meet the international standards of ethics in organ transplantation and informed donor consent. Li Ling, Jinsheng Zeng and Zhijian Liang agree with this retraction. Xiaoshun He has not responded to correspondence from the Publisher about this retraction.
